# Factors That Predict Biological Aggressiveness in Estrogen Receptor–Positive / Human Epidermal Growth Factor Receptor 2–Negative / Lymph Node–Negative Breast Cancer

**DOI:** 10.31486/toj.20.0035

**Published:** 2020

**Authors:** Lauren E. Arthur, Ashley H. McMann, Lauren N. Slattery, George M. Fuhrman, Aimee M. Mackey, Amy E. Rivere, Ralph L. Corsetti

**Affiliations:** ^1^ The University of Queensland Faculty of Medicine, Ochsner Clinical School, New Orleans, LA; ^2^ Department of Surgery, Lieselotte Tansey Breast Center, Ochsner Clinic Foundation, New Orleans, LA; ^3^ Department of Surgery, Division of Surgical Oncology, Tulane University School of Medicine, Covington, LA

**Keywords:** *Breast neoplasms*, *immunohistochemistry*, *neoplasm staging*, *receptors–estrogen*, *receptors–progesterone*, *recurrence*, *sentinel lymph node*

## Abstract

**Background:** Traditionally, breast cancer is staged using TNM criteria: tumor size (T), nodal status (N), and metastasis (M). The Oncotype DX assay provides a recurrence score (RS) based on genomics that predicts the likelihood of distant recurrence in estrogen receptor–positive (ER+)/human epidermal growth factor receptor 2–negative (HER2–)/lymph node–negative (LN–) tumors.

**Methods:** We retrospectively reviewed the medical records of patients with ER+/HER2–/LN– breast cancer tumors who were evaluated between 2007 and 2017 with Oncotype DX RS. We compared the RS to tumor size, patient age, progesterone receptor (PR) status, and LN immunohistochemistry to assess for factors that may independently predict recurrence risk. We also compared tumor size to tumor grade.

**Results:** The data set included 296 tumors: 248 ER+/PR-positive (PR+)/HER2– and 48 ER+/PR-negative (PR–)/HER2–. RS ranged from 0 to 66, patient age ranged from 33 to 77 years, and tumor size ranged from 1 to 65 mm. No significant correlation was found between age and RS (*r*=–0.073, *P*=0.208). PR– tumors had a significantly higher RS regardless of size (PR– mean RS 30.8 ± 12.7; PR+ mean RS 16.3 *±* 7.3; *t*(53)=7.6, *P*<0.0001). No significant correlation was seen between tumor size and RS for all tumors (*r*=–0.028, *P*=0.635), and this finding remained true for the PR+ tumor subgroup (*r*=0.114, *P*=0.072). However, a significant negative correlation was seen between tumor size and RS in the PR– subgroup (*r*=–0.343, *P*=0.017). Further analysis to ensure that differences in tumor grade did not account for this correlation showed equal distribution of well differentiated, moderately differentiated, and poorly differentiated tumors with no significant correlation between tumor size and grade.

**Conclusion:** Increasing tumor size may not be associated with increasing biological aggressiveness. Traditionally, smaller tumors are thought to be lower risk and larger tumors higher risk, with a tendency to use chemotherapy with large tumors. However, our data showed a negative correlation between tumor size and RS in the PR– subgroup. A tumor with PR negativity that reaches a large size without metastasizing may suggest a favorable tumor biology. These tumors may not receive as much benefit from chemotherapy as previously thought. Also, the higher RS seen in smaller PR– tumors may demonstrate PR– status as a predictor for higher risk of distant recurrence. We propose that all tumors meeting the ER+/PR–/LN– criteria, regardless of size, should be considered for genotyping, with the RS used to guide chemotherapy benefit.

## INTRODUCTION

Breast cancer is the most common non–skin cancer worldwide. Approximately 1 in 8 women will be diagnosed with invasive breast cancer in their lifetimes, accounting for an estimated 276,480 women in 2020 alone. Thanks to early screening measures, approximately 64% of newly diagnosed patients are lymph node negative (LN–) without obvious metastatic disease. These screening measures have led to a decline in breast cancer mortality; however, 1 in 39 women will ultimately die from this diagnosis.^1^ Most breast cancers express the estrogen receptor (ER) and/or the progesterone receptor (PR) and do not express the human epidermal growth factor receptor 2 (HER2) across all races/ethnicities. Women with early-stage breast cancer have historically received chemotherapy, endocrine therapy, radiation, surgery, or some combination of these treatments. Risks for distant and/or locoregional recurrence and possible survival benefits are the guiding benchmarks for deciding further therapy.^2^

The Oncotype DX genomic assay is used to help guide decisions about adjuvant chemotherapy. The assay generates a recurrence score (RS) based on the expression of 16 cancer-related genes related to the expression of 5 reference genes. The RS categories are low risk (<18), intermediate risk (18-30), and high risk (>30). The Oncotype DX breast RS provides level 1, category B evidence determining the benefit of chemotherapy in ER-positive (ER+)/HER2-negative (HER2–)/LN– tumors.^3^ Consistent results across multiple well-designed studies have demonstrated the robust analytic performance, clinical validity, and clinical utility of the Oncotype DX assay, and it has been incorporated into multiple guidelines, including those from the National Comprehensive Cancer Network, American Society of Clinical Oncology, National Institute for Health and Care Excellence, St Gallen International Expert Consensus, and European Society for Medical Oncology.^4-8^ The American Joint Committee on Cancer (AJCC) eighth edition cancer staging manual, updated in 2017, uses 2 staging systems: the anatomic stage (tumor size [T], nodal status [N], and distant metastasis [M]) and the prognostic stage (tumor grade, hormone receptor [HR], oncogene expression, and multigene panel testing) to predict a patient's outcome. Oncotype DX is the multigene assay used to help refine prognostic information and improve therapy selection and outcomes. Patients with HR-positive/HER2–/LN– tumors with a low-risk Oncotype DX RS (RS <11) are placed in the same prognostic category as patients with T1a-T1b N0 M0 tumors, meaning all tumors meeting these criteria, regardless of size, are downstaged to stage I.^9^ The low-risk score was lowered to 11 from 18 in the AJCC manual, based on the scale used in the Trial Assigning IndividuaLized Options for Treatment (Rx) (TAILORx) trial in an effort to not undertreat breast cancer.^10^

Historically, tumor size has played a role in staging breast cancer, with larger tumors thought to have a worse prognosis. In a series of 2,282 women with invasive breast cancer or ductal carcinoma in situ, increasing tumor size correlated with lymphatic spread of disease, with percentage rates of LN involvement as follows: Tis, 0.8%; T1a, 5%; T1b, 16%; T1c, 28%; T2, 47%; T3, 68%; and T4, 86%.^11^ These data are reflected in the standard TNM staging.

Other factors that can play a role in the aggressiveness of tumors are patient age, PR status, tumor grade, and further classification of LN– status. Tumor grade is based on how abnormal the cells look under a microscope. Three cancer cell features are evaluated and given a score from 1 to 3. The scores are tallied to assign a grade of 1 (score of 3 to 5), 2 (score of 6 to 7), or 3 (score of 8 to 9) on the pathology report. The terms well differentiated, moderately differentiated, and poorly differentiated are sometimes used in place of the 3 grades. In a study of >15,000 patients, PR status was recognized as an independent factor in predicting responsiveness to endocrine therapy, with benefit in disease-free and overall survival.^12^ In patients with LN– disease, LN status can be further classified as immunohistochemistry (IHC)-negative (i–) or IHC-positive (i+). An i+ status represents an LN that contains malignant cells in clusters ≤0.2 mm and ≤200 total malignant cells on pathology, whereas i– indicates an LN with no malignant cells on pathology. Little research has been conducted to determine the significance of i+ vs i– disease. The Oncotype DX report is not validated for use in LN-positive (LN+) cancers.

The goal of our study was to compare RS scores to tumor size, patient age, PR status, and LN IHC to assess for an independent predictor of recurrence risk. We also compared tumor size with tumor grade.

## METHODS

After receiving institutional review board approval for a retrospective medical records review, we obtained data from the electronic medical record and the Genomic Health Physician Portal for patients with ER+/HER2–/LN– breast cancer who were seen at Ochsner Health between 2007 and 2017 and had an Oncotype DX report. The Oncotype DX RS was used as a marker for biological aggressiveness and to identify factors that may predict increased biological aggressiveness in ER+/HER2–/PR-positive (PR+) or PR-negative (PR–) tumors. We compared tumor size, patient age, PR status, and LN IHC status to low-, intermediate-, and high-risk RS to assess for an association. We also compared tumor size with tumor grade.

ER, PR, and HER2 status was obtained from the Oncotype DX report. However, the Oncotype DX report did not include receptor status for 4 tumors in the 2006 to 2007 time frame. We determined the receptor status for these 4 tumors from pathologic IHC staining. All 4 tumors were ER+/PR+/HER2–.

Tumor size was recorded from the postoperative pathology report. Two patients received preoperative chemotherapy, so pathologic tumor size was smaller than the initial estimated clinical size for 2 tumors. One of these tumors was PR– and the other was PR+. The patient with the PR– tumor had pathologic complete response, with no tumor cells found on final pathology.

All statistical analysis was performed using SPSS software (IBM Corp). Pearson correlation coefficient (*r*) was used to test for a linear relationship in the comparisons of RS and tumor size and RS and patient age. One-way analysis of variance was used for comparisons with multiple variables (RS vs age and RS vs tumor size). Chi-square test was used when the data were treated as categorical variables (RS vs age, RS vs tumor size, RS vs LN IHC, and tumor size vs tumor grade). A *P* value of <0.05 was considered significant. For our analysis of PR status and i– vs i+ nodes, we compared the mean RS in each group. First, a Shapiro-Wilk test for normality was performed. If the data were not normally nonparametric, a Mann-Whitney *U* test was used (RS vs PR status). If the data were normally nonparametric, an independent sample *t* test was used (RS vs IHC status).

## RESULTS

The data set included 296 total tumors: 248 PR+ and 48 PR–. One tumor was from a male patient, and the remainder were from female patients. The mean age of the patients was 58.5 ± 9.8 years (range, 33 to 77 years), the mean tumor size was 17.5 ± 8.7 (range, 1 to 65 mm), and the mean RS was 18.6 ± 9.9 (range, 0 to 66) for all tumors. Data overall and by PR+ and PR– subgroups are shown in Table 1.

**Table 1.** Baseline Demographic and Tumor Characteristics Overall and by Progesterone Receptor Status

Variable	All Tumors n=296	Progesterone Receptor Positive n=248	Progesterone Receptor Negative n=48
Age, years, mean ± SD (range)	58.5 ± 9.8 (33-77)	58.0 ± 9.8 (35-76)	61.3 ± 9.1 (35-77)
Tumor size, mm, mean ± SD (range)	17.5 ± 8.7 (1-65)	17.8 ± 8.8 (1-65)	16.3 ± 8.1 (1-45)
Oncotype DX recurrence score, mean ± SD (range)	18.6 ± 9.9 (0-66)	16.3 ± 7.3 (0-48)	30.8 ± 12.7 (15-66)

As explained in the Methods section, we obtained receptor status from the Oncotype DX report. The Oncotype DX receptor status was concordant with the receptor status in the pathology report for 98% of ER, 85.8% of PR, and 99.7% of HER2 receptors.

### Patient Age vs Recurrence Score

**Figure 1.**
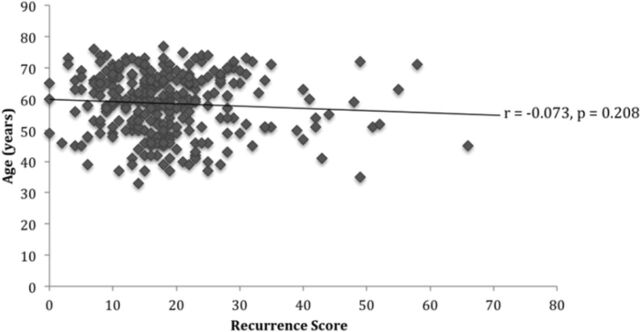
**Age vs recurrence score for estrogen receptor–positive/human epidermal growth factor receptor 2–negative/lymph node–negative breast cancer.** *r*, Pearson correlation coefficient.

As shown in Figure 1, no significant correlation was found between RS and patient age at diagnosis (*r*=–0.073, *P*=0.208). Table 2 shows no significant difference between mean age (*P*=0.489) or age distribution (*P*=0.752) and RS risk category.

**Table 2.** Demographic and Tumor Characteristics by Recurrence Score Category

	Oncotype DX Recurrence Score Category			
Variable	Low Risk (<18) n=152	Intermediate Risk (18-30) n=117	High Risk (>30) n=27	*P* value
Age, years				
Mean ± SD	59 ± 9	58 ± 10	56 ± 10	0.489^a^
Distribution, n (%)				0.752^b^
<40 (n=11)	5 (45.5)	5 (45.5)	1 (9.1)	
40-49 (n=50)	23 (46.0)	23 (46.0)	4 (8.0)	
50-59 (n=85)	44 (51.8)	30 (35.3)	11 (12.9)	
≥60 (n=150)	80 (53.3)	59 (39.3)	11 (7.3)	
Tumor size, mm				
Mean ± SD	1.75 ± 0.93	1.77 ± 0.81	1.68 ± 0.77	0.874^a^
Categorical, n (%)				0.427^b^
<10 (n=36)	19 (52.8)	12 (33.3)	5 (13.9)	
11-20 (n=192)	102 (53.1)	75 (39.1)	15 (7.8)	
21-40 (n=58)	24 (41.4)	27 (46.6)	7 (12.1)	
>40 (n=10)	7 (70.0)	3 (30)	0 (0)	
Progesterone receptor				
Categorical, n (%)				<0.0001^c^
Positive (n=248)	148 (59.7)	92 (37.1)	8 (3.2)	
Negative (n=48)	4 (8.3)	25 (52.1)	19 (39.6)	
Continuous RS, mean ± SD				<0.0001^c^
Positive	16.3 ± 7.3			
Negative	30.8 ± 12.7			
Lymph node immunohistochemistry				
Categorical, n (%)				0.102^b^
Negative (n=263)	131 (49.8)	105 (39.9)	27 (10.3)	
Positive (n=33)	21 (63.6)	12 (36.4)	0 (0)	
Continuous RS, mean ± SD				0.358^d^
Negative	18.9 ± 10.3			
Positive	16.6 ± 6.2			

^a^One-way analysis of variance.

^b^Chi-square test.

^c^Mann-Whitney *U* test.

^d^Independent sample *t* test.

### Tumor Size vs Recurrence Score

**Figure 2.**
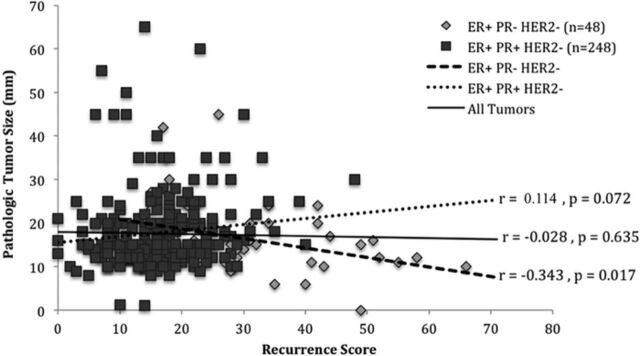
**Pathologic tumor size (mm) vs recurrence score for estrogen receptor–positive (ER+)/human epidermal growth factor receptor 2–negative (HER2–)/lymph node–negative breast cancer.** PR–, progesterone receptor negative; PR+, progesterone receptor positive; *r*, Pearson correlation coefficient.

We compared tumor size and RS for the entire data set and for the 2 subgroups of PR+ and PR– tumors (Figure 2). Pearson correlation coefficient for the entire data set was *r*=–0.028 (*P*=0.635). Analysis of the PR+ subgroup revealed no significant correlation between tumor size and RS (*r*=0.114, *P*=0.072). However, for the PR– subgroup, we found a significant negative correlation between tumor size and RS (*r*=–0.343, *P*=0.017). Smaller tumors were associated with a higher RS. No significant differences were found in the distribution across RS categories for mean tumor size (*P*=0.874) or categorical tumor size (*P*=0.427) (Table 2).

### Tumor Size vs Tumor Grade

To determine if differences in tumor grade accounted for the correlation between tumor size and RS, we compared the mean tumor sizes for each tumor grade (1, 2, or 3). The mean size for well-differentiated tumors was 17.5 mm, for moderately differentiated tumors was 16.3 mm, and for poorly differentiated tumors was 15.0 mm. No statistically significant difference was found (*P*=0.895; data not shown).

### Progesterone Receptor Status vs Recurrence Score

The 248 PR+ tumors had a mean RS of 16.3, and the 48 PR– tumors had a mean RS of 30.8 (Table 1). We found statistical significance between PR status and RS when RS was analyzed as either a categorical or a continuous variable (*P*<0.0001 in both analyses) (Table 2).

### Immunohistochemistry Positive vs Immunohistochemistry Negative

Thirty-three of the LN– tumors had i+ stain for isolated tumor cells. We found no difference between i+ and i– in the analysis of mean continuous RS (*P*=0.358) or in the analysis of RS as a categorical variable (*P*=0.102) (Table 2).

## DISCUSSION

Traditionally, smaller tumors were thought to be lower risk and larger tumors higher risk, as reflected in the current staging of breast cancer with the TNM criteria. According to our data, however, this notion may not be correct for all subtypes of breast cancer. Some subsets of tumors may warrant genomic analysis to more accurately assess the risk of distant recurrence, regardless of initial size at diagnosis.

Evaluation of the relationship between RS and tumor size has been studied multiple times since the validation of the Oncotype DX assay, and a large amount of variation has been seen.^13-20^ The methods by which size and RS, or rate of distant recurrence, are compared throughout these studies differ. In our study, we found no significant correlation between tumor size and RS or any statistically significant difference in mean tumor size between ER+/PR+ or PR–/HER2–/LN– tumors. The prospective trial by Sparano et al found no significant difference in tumor size between low-risk and intermediate-risk cohorts; they did not compare tumor size for the high-risk cohort.^17^ The retrospective review by Hanna et al compared mean tumor sizes for all recurrence risk ranges and found no difference in tumor size between low-risk, intermediate-risk, and high-risk RS.^15^ A prospective study by Paik et al (2004) found no significant correlation between tumor size and distant recurrence when analyzed using a multivariate Cox model. For this comparison, tumors were grouped into 2 sizes: ≤2 cm and >2 cm.^16^ Goldstein et al included both LN– and 0 to 3 LN+ patients in their study. They found no increase in recurrence for tumors ≤2 cm, 2.1 to 5.0 cm, and >2 cm using a proportional hazard model.^14^ Allison et al compared tumors ≤1 cm to tumors >1 cm for all 3 RS risk groups and found no difference.^13^

Three studies report opposing results. Wu et al,^18^ Paik et al (2006),^19^ and Habel et al^20^ compared RS and tumor size and found a significant increase in RS as tumor size increased. The distribution of low-, intermediate-, and high-risk RS varied between our study and these studies. In our study, 9% of the tumors had high-risk RS compared to 25.2% in Paik et al (2006), 31.5% in Habel et al, and 24.6% in Wu et al. This difference in distribution of RS may account for the difference in findings. However, Paik et al (2004) supported our data, and 27% of tumors in that study had high-risk RS.^16^

Another factor that could have led to these conflicting results is the way in which the data were analyzed. Some studies used size and RS as continuous variables and assessed for correlation, while other studies grouped the variables into categories and then assessed for differences. The categories for RS were standard (low, intermediate, and high), but the categories for tumor size varied, with some studies comparing ≤1 cm to >1 cm, comparing ≤2 cm to >2 cm, or using multiple categories. We analyzed the data both ways and found no difference between the continuous or categorical analyses.

A novel aspect of our study is that to our knowledge we are the first to evaluate the difference between the PR+ and PR– subgroups when assessing for a correlation between size and RS (Figure 2). In the PR+ subgroup assessment, a slight positive correlation almost reached significance. In the PR– subgroup assessment, the moderate negative correlation between tumor size and RS reached significance. The lack of correlation or difference in RS in the analysis of our full tumor data set may be attributable to the positive correlation in the PR+ tumors offsetting the negative correlation in the PR– tumors. The negative correlation we found for PR– tumors may be because the larger PR– tumors have a less aggressive tumor biology with less propensity to metastasize compared to the smaller PR– tumors that may have a more aggressive tumor biology with a propensity to metastasize if given time to reach a similar size. This difference in tumor biology of small PR–/LN– tumors and large PR–/LN– tumors may be somewhat elucidated by the specific genes tested in the Oncotype DX genomic panel and would account for the higher RS in small PR–/LN– tumors compared to the lower RS in large PR–/LN– tumors. As stated previously, RS is not validated for use with LN+ tumors; therefore, more aggressive, larger PR– tumors that have already metastasized would not be included in our particular data set. Our study is also limited in that both groups were unequally balanced, with 248 PR+ tumors and 48 PR– tumors.

Younger patients are thought to have more aggressive tumors and are therefore more likely to receive chemotherapy. Our results showed no correlation between age and RS or difference in mean age for each risk group. Our results are in line with the findings of studies by Allison et al, Paik et al (2004), and Wu et al. Allison et al compared mean ages for each risk group and found no significant difference.^13^ Using a multivariate Cox model, Paik et al (2004) assessed the likelihood of distant recurrence in patients <50 years vs patients ≥50 years and found no significant relationship.^16^ Wu et al compared RS in patients ≤55 years and >55 years, finding no significant difference between these groups.^18^

On the other hand, 4 other groups did find a significant difference or correlation between age and recurrence risk. Goldstein et al performed a proportional hazards model for recurrence and found younger age was associated with higher risk of recurrence.^14^ Paik et al (2006) reported a modest concordance between RS and age, with younger patients having a higher RS than older patients.^19^ Levine et al reported a weak correlation between age and RS.^21^ Sparano et al reported a statistically significant but numerically modest difference between low- and intermediate-risk scores in patients with median ages of 58 years and 55 years, respectively.^17^ On further multivariate analysis with tumor grade and age categorically evaluated with ranges of <50 years, 51 years to 60 years, and 61 years to 75 years, no significant association between age and the rate of recurrence was found. In our study, only 11 patients (3.7%) were <40 years old, so this age group was likely underpowered and may account for why we did not see an association between RS and age. We likely did not have enough patients in our data set in the <40 years age range because of our exclusion of LN+ patients, as younger women are more likely to present with nodal disease at diagnosis.^22^ In contrast, approximately 25% of patients in the Goldstein et al study were <45 years.^14^

Patients with breast cancer who are <35 years typically have more aggressive tumor biology and worse prognostic outcomes compared to patients ≥35 years. Multivariate analysis by Albain et al confirmed young age as an independent adverse predictor, finding that cancers in younger patients had significantly higher S-phase fractions and abnormal p53 signaling.^22^ Younger patients also tend to have factors associated with worse prognosis, including grade 3 histology, lymphovascular invasion, necrosis, and ER negativity.^23^

In our study, PR status had a significant association with RS, with PR– tumors having a higher RS than PR+ tumors. The inverse relationship between PR negativity and RS has been reported in multiple studies^13,15,17-19,24^ and may be because PR– tumors are associated with more aggressive features such as higher histologic grade and may be due to tamoxifen resistance from higher expression of HER1 and HER2.^25,26^

We also assessed tumors with i+ nodes vs tumors with i– nodes and found no significant difference in RS. This factor has not been evaluated as fully as tumor size and patient age. The 2 studies that included an assessment of IHC are Allison et al and Hanna et al.^13,15^ Neither study found a difference in RS between i+ and i– nodes.

## CONCLUSION

Increasing tumor size may not be always be associated with increasing biologic aggressiveness. A PR– tumor that has reached a large size without having metastasized likely has a favorable tumor biology that may not necessarily benefit as much as previously thought from chemotherapy. Based on our results, we propose that all tumors meeting the ER+/PR–/LN– criteria, regardless of size, be considered for genotyping. This recommendation is supported by the negative correlation seen in the PR– subgroup analysis of tumor size and RS. The significantly higher RS in PR– tumors demonstrates PR– receptor status as an independent predictor for higher risk of distant recurrence.

## Notes

This article meets the Accreditation Council for Graduate Medical Education and the American Board of Medical Specialties Maintenance of Certification competencies for Patient Care, Medical Knowledge, and Practice-Based Learning and Improvement.
